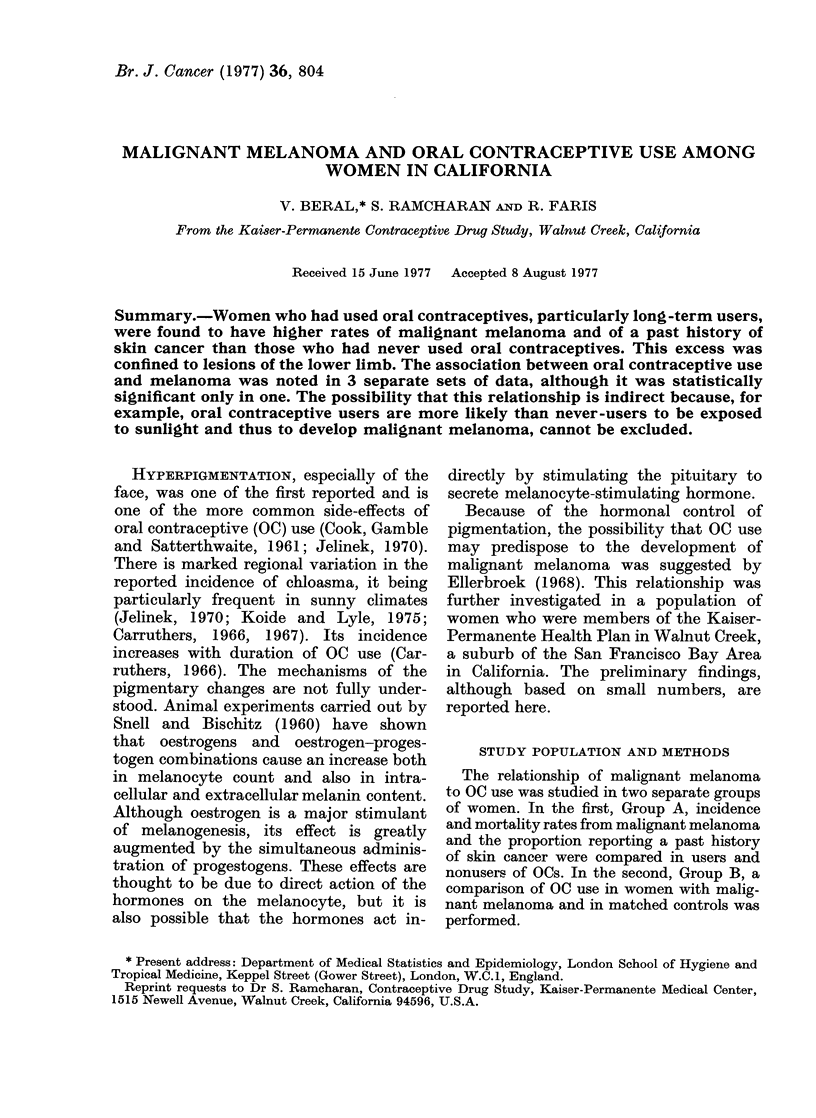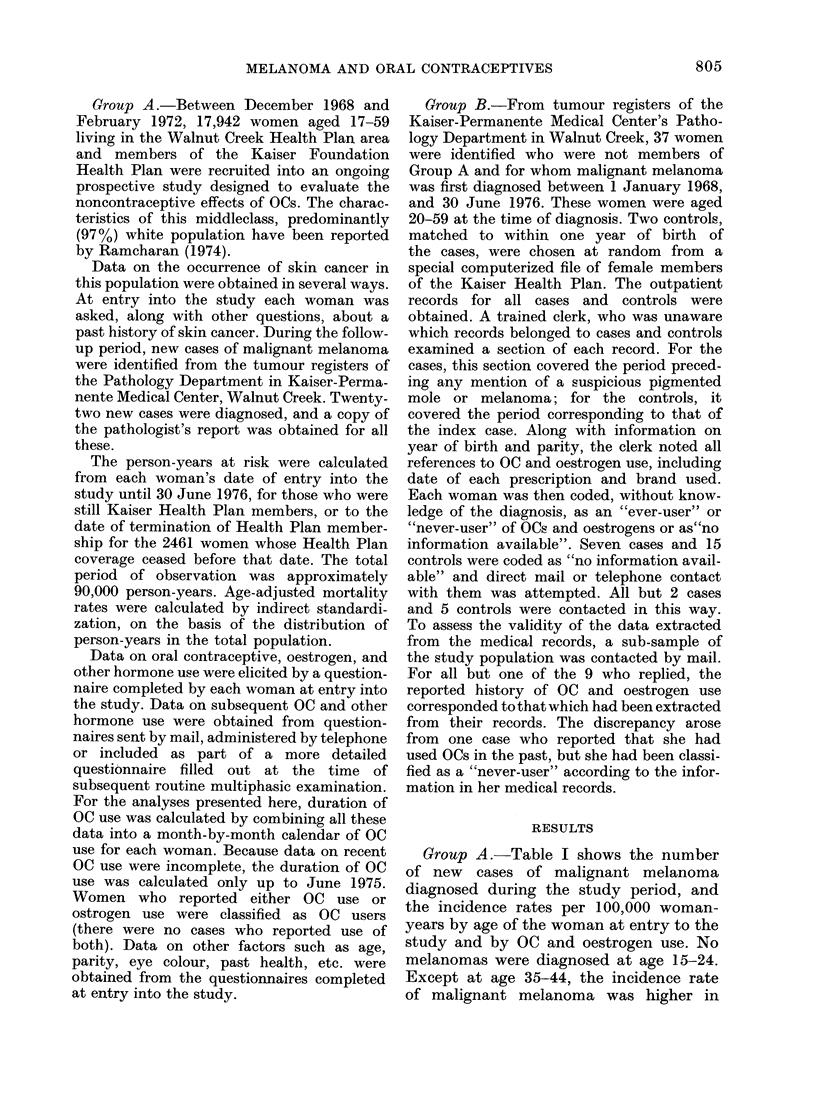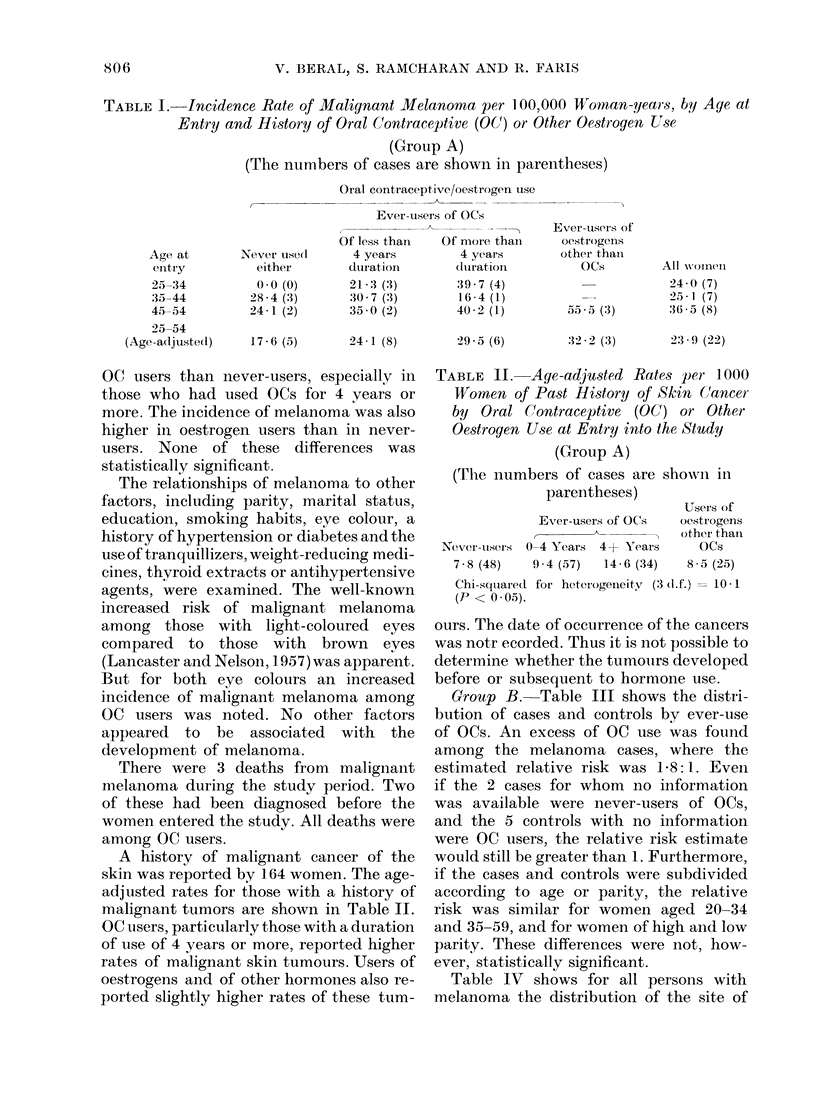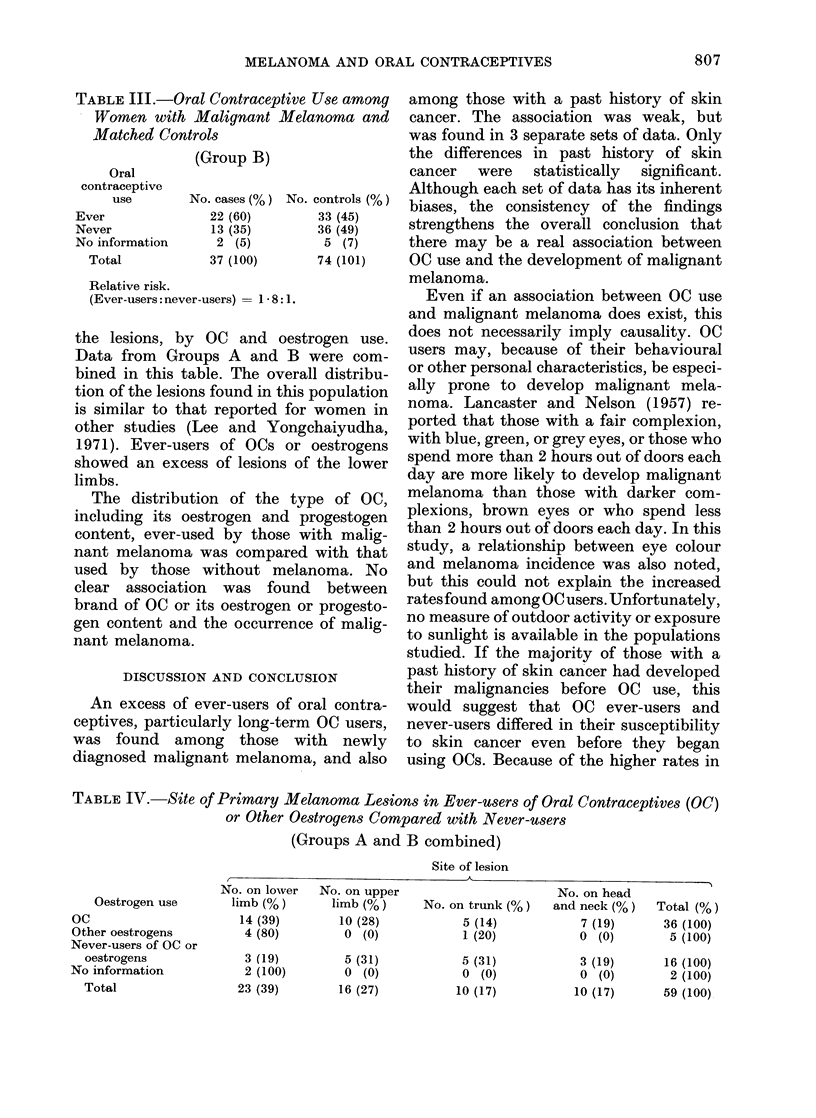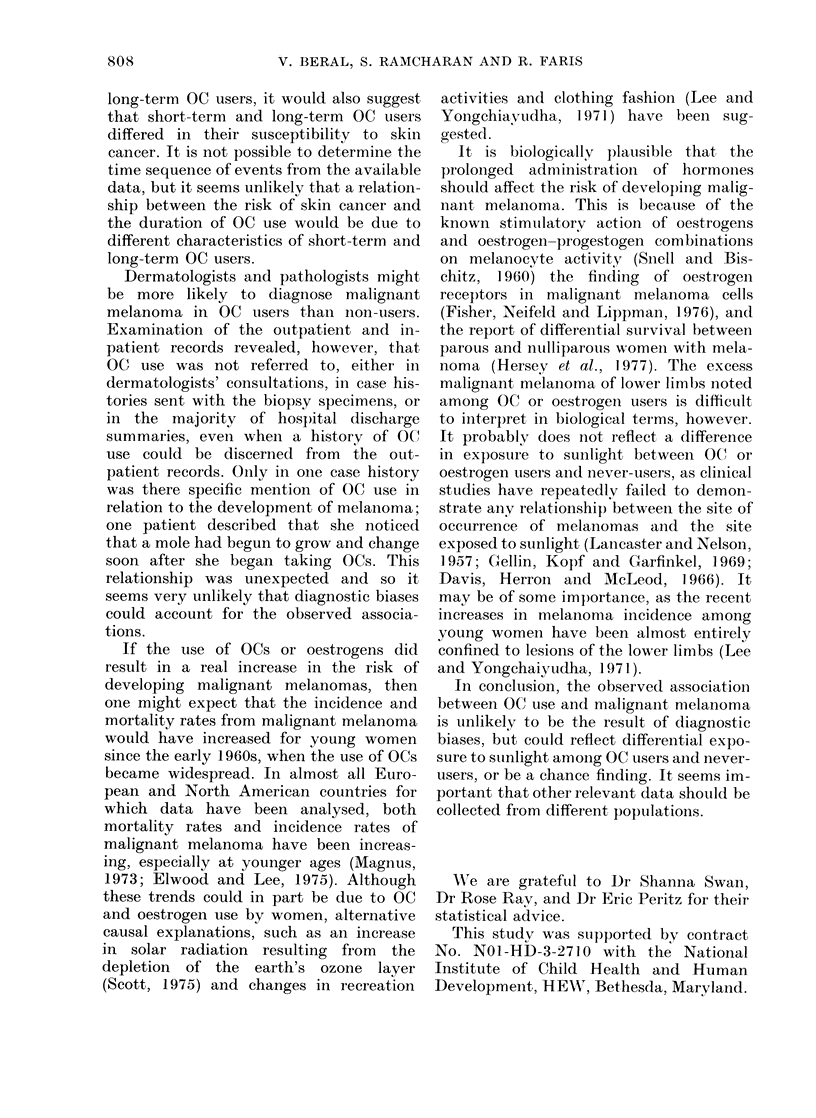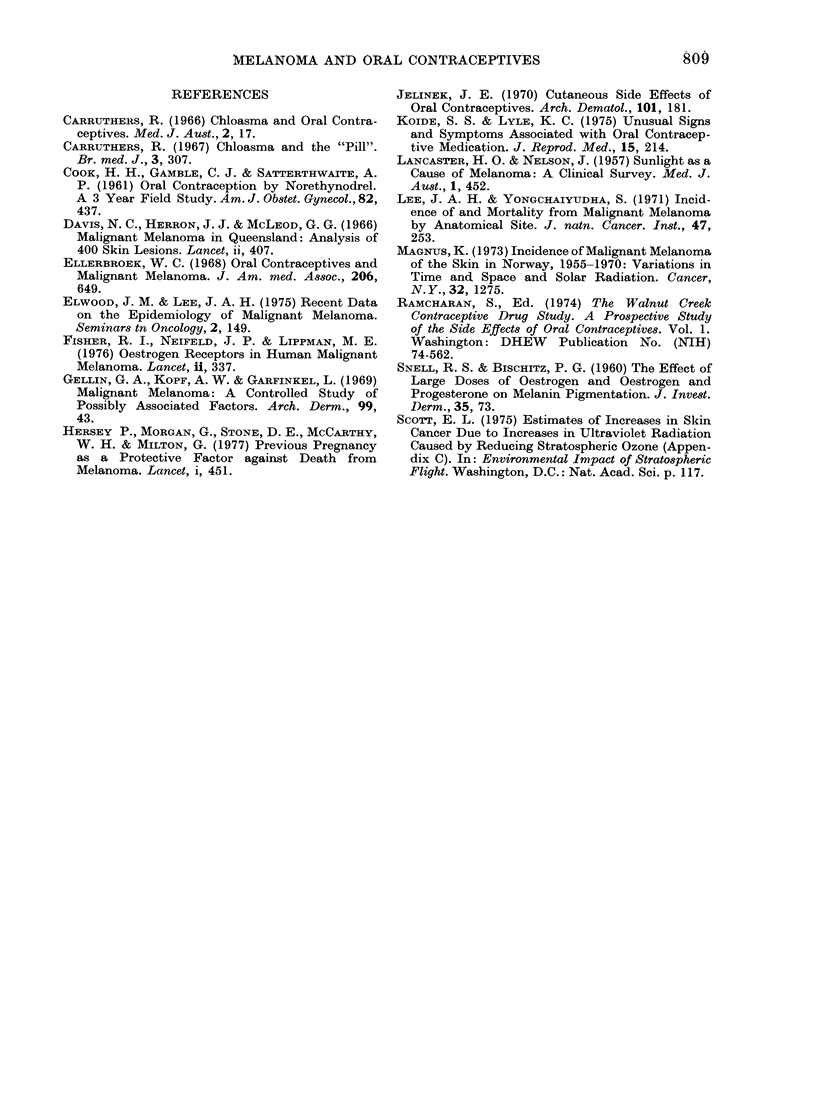# Malignant melanoma and oral contraceptive use among women in California.

**DOI:** 10.1038/bjc.1977.265

**Published:** 1977-12

**Authors:** V. Beral, S. Ramcharan, R. Faris

## Abstract

Women who had used oral contraceptives, particularly long-term users, were found to have higher rates of malignant melanoma and of a past history of skin cancer than those who had never used oral contraceptives. This excess was confined to lesions of the lower limb. The association between oral contraceptive use and melanoma was noted in 3 separate sets of data, although it was statistically significant only in one. The possibility that this relationship is indirect because, for example, oral contraceptive users are more likely than never-users to be exposed to sunlight and thus to develop malignant melanoma, cannot be excluded.


*Br. J. Cancer* (1977) **36**, 804

### MALIGNANT MELANOMA AND ORAL CONTRACEPTIVE USE AMONG WOMEN IN CALIFORNIA

V. BERAL,* S. RAMCHARAN AND R. FARIS

*From the Kaiser-Permanente Contraceptive Drug Study, Walnut Creek, California*

Received 15 June 1977 Accepted 8 August 1977

**Summary.—Women who had used oral contraceptives, particularly long-term users, were found to have higher rates of malignant melanoma and of a past history of skin cancer than those who had never used oral contraceptives. This excess was confined to lesions of the lower limb. The association between oral contraceptive use and melanoma was noted in 3 separate sets of data, although it was statistically significant only in one. The possibility that this relationship is indirect because, for example, oral contraceptive users are more likely than never-users to be exposed to sunlight and thus to develop malignant melanoma, cannot be excluded.**

HYPERPIGMENTATION, especially of the face, was one of the first reported and is one of the more common side-effects of oral contraceptive (OC) use (Cook, Gamble and Satterthwaite, 1961; Jelinek, 1970). There is marked regional variation in the reported incidence of chloasma, it being particularly frequent in sunny climates (Jelinek, 1970; Koide and Lyle, 1975; Carruthers, 1966, 1967). Its incidence increases with duration of OC use (Carruthers, 1966). The mechanisms of the pigmentary changes are not fully understood. Animal experiments carried out by Snell and Bischitz (1960) have shown that oestrogens and oestrogen–progestogen combinations cause an increase both in melanocyte count and also in intracellular and extracellular melanin content. Although oestrogen is a major stimulant of melanogenesis, its effect is greatly augmented by the simultaneous administration of progestogens. These effects are thought to be due to direct action of the hormones on the melanocyte, but it is also possible that the hormones act indirectly by stimulating the pituitary to secrete melanocyte-stimulating hormone.

Because of the hormonal control of pigmentation, the possibility that OC use may predispose to the development of malignant melanoma was suggested by Ellerbroek (1968). This relationship was further investigated in a population of women who were members of the Kaiser-Permanente Health Plan in Walnut Creek, a suburb of the San Francisco Bay Area in California. The preliminary findings, although based on small numbers, are reported here.

### STUDY POPULATION AND METHODS

The relationship of malignant melanoma to OC use was studied in two separate groups of women. In the first, Group A, incidence and mortality rates from malignant melanoma and the proportion reporting a past history of skin cancer were compared in users and nonusers of OCs. In the second, Group B, a comparison of OC use in women with malignant melanoma and in matched controls was performed.

* Present address: Department of Medical Statistics and Epidemiology, London School of Hygiene and Tropical Medicine, Keppel Street (Gower Street), London, W.C.1, England.

Reprint requests to Dr S. Ramcharan, Contraceptive Drug Study, Kaiser-Permanente Medical Center, 1515 Newell Avenue, Walnut Creek, California 94596, U.S.A.

*Group A*.—Between December 1968 and February 1972, 17,942 women aged 17–59 living in the Walnut Creek Health Plan area and members of the Kaiser Foundation Health Plan were recruited into an ongoing prospective study designed to evaluate the noncontraceptive effects of OCs. The characteristics of this middleclass, predominantly (97%) white population have been reported by Ramcharan (1974).

Data on the occurrence of skin cancer in this population were obtained in several ways. At entry into the study each woman was asked, along with other questions, about a past history of skin cancer. During the follow-up period, new cases of malignant melanoma were identified from the tumour registers of the Pathology Department in Kaiser-Permanente Medical Center, Walnut Creek. Twenty-two new cases were diagnosed, and a copy of the pathologist's report was obtained for all these.

The person-years at risk were calculated from each woman's date of entry into the study until 30 June 1976, for those who were still Kaiser Health Plan members, or to the date of termination of Health Plan membership for the 2461 women whose Health Plan coverage ceased before that date. The total period of observation was approximately 90,000 person-years. Age-adjusted mortality rates were calculated by indirect standardization, on the basis of the distribution of person-years in the total population.

Data on oral contraceptive, oestrogen, and other hormone use were elicited by a questionnaire completed by each woman at entry into the study. Data on subsequent OC and other hormone use were obtained from questionnaires sent by mail, administered by telephone or included as part of a more detailed questionnaire filled out at the time of subsequent routine multiphasic examination. For the analyses presented here, duration of OC use was calculated by combining all these data into a month-by-month calendar of OC use for each woman. Because data on recent OC use were incomplete, the duration of OC use was calculated only up to June 1975. Women who reported either OC use or ostrogen use were classified as OC users (there were no cases who reported use of both). Data on other factors such as age, parity, eye colour, past health, etc. were obtained from the questionnaires completed at entry into the study.

*Group B*.—From tumour registers of the Kaiser-Permanente Medical Center's Pathology Department in Walnut Creek, 37 women were identified who were not members of Group A and for whom malignant melanoma was first diagnosed between 1 January 1968, and 30 June 1976. These women were aged 20–59 at the time of diagnosis. Two controls, matched to within one year of birth of the cases, were chosen at random from a special computerized file of female members of the Kaiser Health Plan. The outpatient records for all cases and controls were obtained. A trained clerk, who was unaware which records belonged to cases and controls examined a section of each record. For the cases, this section covered the period preceding any mention of a suspicious pigmented mole or melanoma; for the controls, it covered the period corresponding to that of the index case. Along with information on year of birth and parity, the clerk noted all references to OC and oestrogen use, including date of each prescription and brand used. Each woman was then coded, without knowledge of the diagnosis, as an "ever-user" or "never-user" of OCs and oestrogens or as"no information available". Seven cases and 15 controls were coded as "no information available" and direct mail or telephone contact with them was attempted. All but 2 cases and 5 controls were contacted in this way. To assess the validity of the data extracted from the medical records, a sub-sample of the study population was contacted by mail. For all but one of the 9 who replied, the reported history of OC and oestrogen use corresponded to that which had been extracted from their records. The discrepancy arose from one case who reported that she had used OCs in the past, but she had been classified as a "never-user" according to the information in her medical records.

### RESULTS

*Group A*.—Table I shows the number of new cases of malignant melanoma diagnosed during the study period, and the incidence rates per 100,000 woman-years by age of the woman at entry to the study and by OC and oestrogen use. No melanomas were diagnosed at age 15–24. Except at age 35–44, the incidence rate of malignant melanoma was higher in

TABLE I.—*Incidence Rate of Malignant Melanoma per 100,000 Woman-years, by Age at Entry and History of Oral Contraceptive (OC) or Other Oestrogen Use*

(Group A)

(The numbers of cases are shown in parentheses)

| | Oral contraceptive/oestrogen use | | | | |
|---|---|---|---|---|---|
| | | Ever-users of OCs | | | |
| Age at entry | Never used either | Of less than 4 years duration | Of more than 4 years duration | Ever-users of oestrogens other than OCs | All women |
| 25–34 | 0·0 (0) | 21·3 (3) | 39·7 (4) | — | 24·0 (7) |
| 35–44 | 28·4 (3) | 30·7 (3) | 16·4 (1) | — | 25·1 (7) |
| 45–54 | 24·1 (2) | 35·0 (2) | 40·2 (1) | 55·5 (3) | 36·5 (8) |
| 25–54 (Age-adjusted) | 17·6 (5) | 24·1 (8) | 29·5 (6) | 32·2 (3) | 23·9 (22) |

OC users than never-users, especially in those who had used OCs for 4 years or more. The incidence of melanoma was also higher in oestrogen users than in never-users. None of these differences was statistically significant.

The relationships of melanoma to other factors, including parity, marital status, education, smoking habits, eye colour, a history of hypertension or diabetes and the use of tranquillizers, weight-reducing medicines, thyroid extracts or antihypertensive agents, were examined. The well-known increased risk of malignant melanoma among those with light-coloured eyes compared to those with brown eyes (Lancaster and Nelson, 1957) was apparent. But for both eye colours an increased incidence of malignant melanoma among OC users was noted. No other factors appeared to be associated with the development of melanoma.

There were 3 deaths from malignant melanoma during the study period. Two of these had been diagnosed before the women entered the study. All deaths were among OC users.

A history of malignant cancer of the skin was reported by 164 women. The age-adjusted rates for those with a history of malignant tumors are shown in Table II. OC users, particularly those with a duration of use of 4 years or more, reported higher rates of malignant skin tumours. Users of oestrogens and of other hormones also reported slightly higher rates of these tumours. The date of occurrence of the cancers was notr ecorded. Thus it is not possible to determine whether the tumours developed before or subsequent to hormone use.

TABLE II.—*Age-adjusted Rates per 1000 Women of Past History of Skin Cancer by Oral Contraceptive (OC) or Other Oestrogen Use at Entry into the Study*

(Group A)

(The numbers of cases are shown in parentheses)

| | Ever-users of OCs | | Users of oestrogens other than OCs |
|---|---|---|---|
| Never-users | 0–4 Years | 4+ Years | |
| 7·8 (48) | 9·4 (57) | 14·6 (34) | 8·5 (25) |

Chi-squared for heterogeneity (3 d.f.) = 10·1 ($P < 0·05$).

*Group B.*—Table III shows the distribution of cases and controls by ever-use of OCs. An excess of OC use was found among the melanoma cases, where the estimated relative risk was 1·8:1. Even if the 2 cases for whom no information was available were never-users of OCs, and the 5 controls with no information were OC users, the relative risk estimate would still be greater than 1. Furthermore, if the cases and controls were subdivided according to age or parity, the relative risk was similar for women aged 20–34 and 35–59, and for women of high and low parity. These differences were not, however, statistically significant.

Table IV shows for all persons with melanoma the distribution of the site of

TABLE III.—*Oral Contraceptive Use among Women with Malignant Melanoma and Matched Controls*

(Group B)

| Oral contraceptive use | No. cases (%) | No. controls (%) |
|---|---|---|
| Ever | 22 (60) | 33 (45) |
| Never | 13 (35) | 36 (49) |
| No information | 2 (5) | 5 (7) |
| Total | 37 (100) | 74 (101) |

Relative risk.
(Ever-users:never-users) = 1·8:1.

the lesions, by OC and oestrogen use. Data from Groups A and B were combined in this table. The overall distribution of the lesions found in this population is similar to that reported for women in other studies (Lee and Yongchaiyudha, 1971). Ever-users of OCs or oestrogens showed an excess of lesions of the lower limbs.

The distribution of the type of OC, including its oestrogen and progestogen content, ever-used by those with malignant melanoma was compared with that used by those without melanoma. No clear association was found between brand of OC or its oestrogen or progestogen content and the occurrence of malignant melanoma.

### DISCUSSION AND CONCLUSION

An excess of ever-users of oral contraceptives, particularly long-term OC users, was found among those with newly diagnosed malignant melanoma, and also among those with a past history of skin cancer. The association was weak, but was found in 3 separate sets of data. Only the differences in past history of skin cancer were statistically significant. Although each set of data has its inherent biases, the consistency of the findings strengthens the overall conclusion that there may be a real association between OC use and the development of malignant melanoma.

Even if an association between OC use and malignant melanoma does exist, this does not necessarily imply causality. OC users may, because of their behavioural or other personal characteristics, be especially prone to develop malignant melanoma. Lancaster and Nelson (1957) reported that those with a fair complexion, with blue, green, or grey eyes, or those who spend more than 2 hours out of doors each day are more likely to develop malignant melanoma than those with darker complexions, brown eyes or who spend less than 2 hours out of doors each day. In this study, a relationship between eye colour and melanoma incidence was also noted, but this could not explain the increased rates found among OC users. Unfortunately, no measure of outdoor activity or exposure to sunlight is available in the populations studied. If the majority of those with a past history of skin cancer had developed their malignancies before OC use, this would suggest that OC ever-users and never-users differed in their susceptibility to skin cancer even before they began using OCs. Because of the higher rates in

TABLE IV.—*Site of Primary Melanoma Lesions in Ever-users of Oral Contraceptives (OC) or Other Oestrogens Compared with Never-users*

(Groups A and B combined)

| | Site of lesion | | | | |
|---|---|---|---|---|---|
| Oestrogen use | No. on lower limb (%) | No. on upper limb (%) | No. on trunk (%) | No. on head and neck (%) | Total (%) |
| OC | 14 (39) | 10 (28) | 5 (14) | 7 (19) | 36 (100) |
| Other oestrogens | 4 (80) | 0 (0) | 1 (20) | 0 (0) | 5 (100) |
| Never-users of OC or oestrogens | 3 (19) | 5 (31) | 5 (31) | 3 (19) | 16 (100) |
| No information | 2 (100) | 0 (0) | 0 (0) | 0 (0) | 2 (100) |
| Total | 23 (39) | 16 (27) | 10 (17) | 10 (17) | 59 (100) |

long-term OC users, it would also suggest that short-term and long-term OC users differed in their susceptibility to skin cancer. It is not possible to determine the time sequence of events from the available data, but it seems unlikely that a relationship between the risk of skin cancer and the duration of OC use would be due to different characteristics of short-term and long-term OC users.

Dermatologists and pathologists might be more likely to diagnose malignant melanoma in OC users than non-users. Examination of the outpatient and inpatient records revealed, however, that OC use was not referred to, either in dermatologists' consultations, in case histories sent with the biopsy specimens, or in the majority of hospital discharge summaries, even when a history of OC use could be discerned from the outpatient records. Only in one case history was there specific mention of OC use in relation to the development of melanoma; one patient described that she noticed that a mole had begun to grow and change soon after she began taking OCs. This relationship was unexpected and so it seems very unlikely that diagnostic biases could account for the observed associations.

If the use of OCs or oestrogens did result in a real increase in the risk of developing malignant melanomas, then one might expect that the incidence and mortality rates from malignant melanoma would have increased for young women since the early 1960s, when the use of OCs became widespread. In almost all European and North American countries for which data have been analysed, both mortality rates and incidence rates of malignant melanoma have been increasing, especially at younger ages (Magnus, 1973; Elwood and Lee, 1975). Although these trends could in part be due to OC and oestrogen use by women, alternative causal explanations, such as an increase in solar radiation resulting from the depletion of the earth's ozone layer (Scott, 1975) and changes in recreation activities and clothing fashion (Lee and Yongchiayudha, 1971) have been suggested.

It is biologically plausible that the prolonged administration of hormones should affect the risk of developing malignant melanoma. This is because of the known stimulatory action of oestrogens and oestrogen–progestogen combinations on melanocyte activity (Snell and Bischitz, 1960) the finding of oestrogen receptors in malignant melanoma cells (Fisher, Neifeld and Lippman, 1976), and the report of differential survival between parous and nulliparous women with melanoma (Hersey *et al.*, 1977). The excess malignant melanoma of lower limbs noted among OC or oestrogen users is difficult to interpret in biological terms, however. It probably does not reflect a difference in exposure to sunlight between OC or oestrogen users and never-users, as clinical studies have repeatedly failed to demonstrate any relationship between the site of occurrence of melanomas and the site exposed to sunlight (Lancaster and Nelson, 1957; Gellin, Kopf and Garfinkel, 1969; Davis, Herron and McLeod, 1966). It may be of some importance, as the recent increases in melanoma incidence among young women have been almost entirely confined to lesions of the lower limbs (Lee and Yongchaiyudha, 1971).

In conclusion, the observed association between OC use and malignant melanoma is unlikely to be the result of diagnostic biases, but could reflect differential exposure to sunlight among OC users and never-users, or be a chance finding. It seems important that other relevant data should be collected from different populations.

We are grateful to Dr Shanna Swan, Dr Rose Ray, and Dr Eric Peritz for their statistical advice.

This study was supported by contract No. N01-HD-3-2710 with the National Institute of Child Health and Human Development, HEW, Bethesda, Maryland.

### REFERENCES

CARRUTHERS, R. (1966) Chloasma and Oral Contraceptives. *Med. J. Aust.*, **2**, 17.

CARRUTHERS, R. (1967) Chloasma and the "Pill". *Br. med. J.*, **3**, 307.

COOK, H. H., GAMBLE, C. J. & SATTERTHWAITE, A. P. (1961) Oral Contraception by Norethynodrel. A 3 Year Field Study. *Am. J. Obstet. Gynecol.*, **82**, 437.

DAVIS, N. C., HERRON, J. J. & McLEOD, G. G. (1966) Malignant Melanoma in Queensland: Analysis of 400 Skin Lesions. *Lancet*, ii, 407.

ELLERBROEK, W. C. (1968) Oral Contraceptives and Malignant Melanoma. *J. Am. med. Assoc.*, **206**, 649.

ELWOOD, J. M. & LEE, J. A. H. (1975) Recent Data on the Epidemiology of Malignant Melanoma. *Seminars tn Oncology*, **2**, 149.

FISHER, R. I., NEIFELD, J. P. & LIPPMAN, M. E. (1976) Oestrogen Receptors in Human Malignant Melanoma. *Lancet*, **ii**, 337.

GELLIN, G. A., KOPF, A. W. & GARFINKEL, L. (1969) Malignant Melanoma: A Controlled Study of Possibly Associated Factors. *Arch. Derm.*, **99**, 43.

HERSEY P., MORGAN, G., STONE, D. E., McCARTHY, W. H. & MILTON, G. (1977) Previous Pregnancy as a Protective Factor against Death from Melanoma. *Lancet*, i, 451.

JELINEK, J. E. (1970) Cutaneous Side Effects of Oral Contraceptives. *Arch. Dematol.*, **101**, 181.

KOIDE, S. S. & LYLE, K. C. (1975) Unusual Signs and Symptoms Associated with Oral Contraceptive Medication. *J. Reprod. Med.*, **15**, 214.

LANCASTER, H. O. & NELSON, J. (1957) Sunlight as a Cause of Melanoma: A Clinical Survey. *Med. J. Aust.*, **1**, 452.

LEE, J. A. H. & YONGCHAIYUDHA, S. (1971) Incidence of and Mortality from Malignant Melanoma by Anatomical Site. *J. natn. Cancer. Inst.*, **47**, 253.

MAGNUS, K. (1973) Incidence of Malignant Melanoma of the Skin in Norway, 1955–1970: Variations in Time and Space and Solar Radiation. *Cancer, N.Y.*, **32**, 1275.

RAMCHARAN, S., Ed. (1974) *The Walnut Creek Contraceptive Drug Study. A Prospective Study of the Side Effects of Oral Contraceptives.* Vol. 1. Washington: DHEW Publication No. (NIH) 74-562.

SNELL, R. S. & BISCHITZ, P. G. (1960) The Effect of Large Doses of Oestrogen and Oestrogen and Progesterone on Melanin Pigmentation. *J. Invest. Derm.*, **35**, 73.

SCOTT, E. L. (1975) Estimates of Increases in Skin Cancer Due to Increases in Ultraviolet Radiation Caused by Reducing Stratospheric Ozone (Appendix C). In: *Environmental Impact of Stratospheric Flight.* Washington, D.C.: Nat. Acad. Sci. p. 117.